# Crystal structure of *Brugia malayi* venom allergen-like protein-1 (BmVAL-1), a vaccine candidate for lymphatic filariasis

**DOI:** 10.1016/j.ijpara.2017.12.003

**Published:** 2018-04

**Authors:** Rabih Darwiche, Fernanda Lugo, Claire Drurey, Koen Varossieau, Geert Smant, Ruud H.P. Wilbers, Rick M. Maizels, Roger Schneiter, Oluwatoyin A. Asojo

**Affiliations:** a Division of Biochemistry, Department of Biology, University of Fribourg, Chemin du Musée 10, CH 1700 Fribourg, Switzerland; b National School of Tropical Medicine, Baylor College of Medicine, Houston, TX 77030, USA; c Wellcome Centre for Molecular Parasitology, Institute for Infection, Immunity and Inflammation, University of Glasgow, Sir Graeme Davies Building, 120 University Place, Glasgow G12 8TA, UK; d Laboratory of Nematology, Wageningen University, Droevendaalsesteeg 1, 6708 PB Wageningen, The Netherlands

**Keywords:** *Ancylostoma* secreted protein (ASP), Sperm coating protein (SCP), CAP [cysteine-rich secretory protein (CRISP)/antigen 5/pathogenesis related-1 (PR-1)], Venom antigen 5, Excretory-secretory products, Sterol binding

## Abstract

*Brugia malayi* is a causative agent of lymphatic filariasis, a major tropical disease. The infective L3 parasite stage releases immunomodulatory proteins including the venom allergen-like proteins (VALs), which are members of the SCP/TAPS (Sperm-coating protein/Tpx/antigen 5/pathogenesis related-1/Sc7) superfamily. BmVAL-1 is a major target of host immunity with >90% of infected *B. malayi* microfilaraemic cases being seropositive for antibodies to BmVAL-1. This study is part of ongoing efforts to characterize the structures and functions of important *B. malayi* proteins. Recombinant BmVAL-1 was produced using a plant expression system, crystallized and the structure was solved by molecular replacement and refined to 2.1 Å, revealing the characteristic alpha/beta/alpha sandwich topology of eukaryotic SCP/TAPS proteins. The protein has more than 45% loop regions and these flexible loops connect the helices and strands, which are longer than predicted based on other parasite SCP/TAPS protein structures. The large central cavity of BmVAL-1 is a prototypical CRISP cavity with two histidines required to bind divalent cations. The caveolin-binding motif (CBM) that mediates sterol binding in SCP/TAPS proteins is large and open in BmVAL-1 and is N-glycosylated. N-glycosylation of the CBM does not affect the ability of BmVAL-1 to bind sterol in vitro. BmVAL-1 complements the in vivo sterol export phenotype of yeast mutants lacking their endogenous SCP/TAPS proteins. The in vitro sterol-binding affinity of BmVAL-1 is comparable with Pry1, a yeast sterol transporting SCP/TAPS protein. Sterol binding of BmVAL-1 is dependent on divalent cations. BmVAL-1 also has a large open palmitate-binding cavity, which binds palmitate comparably to tablysin-15, a lipid-binding SCP/TAPS protein. The central cavity, CBM and palmitate-binding cavity of BmVAL-1 are interconnected within the monomer with channels that can serve as pathways for water molecules, cations and small molecules.

## Highlights

• The vaccine candidate *Brugia malayi* venom allergen-like 1 protein (BmVAL-1) has three distinct binding cavities.
• The cavities are the central cavity; the sterol-binding caveolin-binding motif (CBM); and the palmitate-binding cavity.
• These cavities are connected by channels, which can accommodate water molecules, ions and small ligands.
• The channels explain how blocking divalent ions in the central cavity affects sterol binding in the distinct CBM cavity.
• BmVAL-1 has a glycosylated CBM, is an effective sterol transporter in vivo and binds cholesterol and palmitate in vitro.

## Graphical abstract

**Figure:**
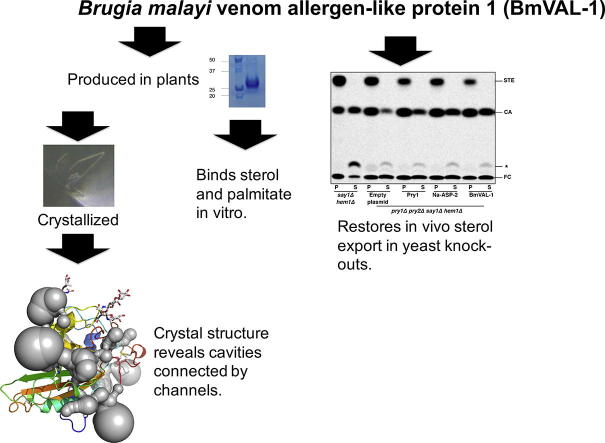

## 1 Introduction

The roundworm *Brugia malayi* is one of the causative agents of lymphatic filariasis that affects over 120 million people in 83 countries in the tropics and subtropics. *Brugia malayi* venom allergen-like protein 1 (BmVAL-1) protein was discovered as a highly expressed transcript representing ∼2% of cDNAs from the mosquito-borne infective larval (L3) stage, and at lower levels in subsequent post-infective mammalian stages (Murray et al., 2001). Expression of BmVAL-1 falls 10-fold once larvae are exposed to mammalian-like conditions in vitro (Li et al., 2009). Immunologically, BmVAL-1 is a major target of host immunity with >90% of infected *B. malayi* microfilaraemic cases being seropositive for the recombinant protein, and evidence from mouse models that the antigen induces a strong T cell response (Murray et al., 2001). Supporting this, T cells from humans infected with the closely related species *Wuchereria bancrofti* respond to the Wb-VAL (>90% identical at amino acid level) with proliferation and cytokine release (Anand et al., 2007). Hence, BmVAL-1 has been considered a potential vaccine antigen, with confirmed serological reactivity in infected humans and a reported 40–50% reduction in adult worm load in jirds (*Meriones unguiculatus*) that had been vaccinated with BmVAL-1 prior to L3 challenge (Kalyanasundaram and Balumuri, 2011).

BmVAL-1 belongs to the eukaryotic CAP (cysteine-rich secretory protein/antigen 5/pathogenesis related-1) or SCP/TAPS (Sperm-coating protein/Tpx/antigen 5/pathogenesis related-1/Sc7) superfamily of proteins, which has been implicated in biological processes such as reproduction, fungal virulence, cellular defense, and immune evasion (Hawdon et al., 1999, Ding et al., 2000, Gao et al., 2001, Zhan et al., 2003, Schneiter and Di Pietro, 2013). SCP/TAPS proteins have been implicated in pathogen defense and in plants the sterol-binding ability is important for protection against a class of plant fungal pathogens known as oomycetes (Gamir et al., 2017). Previous in vitro studies revealed that the inhibition of oomycete growth by recombinant plant CAP protein (PR-1) was likely due to sterol sequestration (Darwiche et al., 2017, Gamir et al., 2017, Kazan and Gardiner, 2017). Specifically, plants overexpressing PR-1 have been shown to be particularly resistant to sterol auxotroph pathogens such as oomycetes. Mutant phenotypes of sterol transport-deficient yeast mutants can be reverted by plant PR-1 isoforms, PR-1 sterol-binding activities correlate with antimicrobial activities and sterol non-auxotrophic fungi can be made susceptible to PR-1 by inhibiting their own sterol biosynthesis. This explains the mode of action of PR-1 in plant immunity and its higher activity against oomycetes, which are unable to synthesize their own sterols. Furthermore, plant PR-1 can sequester leaky sterols from the apoplast, and actively retrieve sterols from oomycete membranes (Gamir et al., 2017).

Similar to VAL proteins from other filarial parasites, BmVAL-1 has a single ∼15 kDa CAP domain. Two ProSITE motifs have been defined for SCP/TAPS proteins CAP1/CRISP1 [GDER][HR][FYWH][TVS][QA][LIVM][LIVMA]Wxx[STN], and CAP2/CRISP2 [LIVMFYH][LIVMFY]xC[NQRHS]Yx[PARH]x[GL]N[LIVMFYWDN (de Castro et al., 2006, Yoon et al., 2007). Two additional motifs, CAP3/CRISP3 (HNxxR) and CAP4/CRISP4 (G[EQ]N[ILV], were defined subsequently (Gibbs et al., 2008). Each motif contributes residues that align the central cavity including two histidines residues from CAP1 and CAP3 that bind divalent cations including Zn^2+^ and Mg^2+^ (Serrano et al., 2004, Asojo et al., 2005, Asojo et al., 2011, Gibbs et al., 2008, Suzuki et al., 2008, Wang et al., 2010, van Galen et al., 2012, Xu et al., 2012, Mason et al., 2014). Although BmVAL-1 does not fully conform to the ProSITE motifs, it contains both characteristic central cavity histidine residues. The amino acid sequence of BmVAL-1 differs from other SCP/TAPS proteins with known structures. The highest sequence identity and coverage is with the human hookworm proteins *Necator americanus Ancylostoma* secreted protein-2, (Na-ASP-2, 37% sequence identity and 97% coverage) and *N. americanus Ancylostoma* secreted protein-1, (Na-ASP-1, 35% sequence identity and 99% coverage), and other structures have less than 85% query coverage (Asojo et al., 2005, Asojo, 2011). Furthermore, previous structural analyses reveal that despite sharing an alpha/beta/alpha sandwich topology, SCP/TAPS proteins are >40% loop regions, making it difficult to accurately model their structures. As part of efforts to characterize the functions of filarial parasite vaccine candidates, recombinant BmVAL-1 was produced and its structure was determined.

## 2 Materials and methods

### 2.1 Plant-based expression of BmVAL-1

The codon optimized sequence encoding mature BmVAL-1, omitting its endogenous 16AA signal peptide, was cloned into a pHYG expression vector downstream from the *Arabidopsis thaliana* chitinase signal peptide (cSP). The BmVAL-1 expression vector was transformed into *Agrobacterium tumefaciens* (strain MOG101) for agro-infiltration and co-infiltrated with the pBIN61 vector containing the silencing inhibitor p19 from tomato bushy stunt virus. BmVAL-1 and p19 *Agrobacterium tumefaciens* clones were grown in Lennox broth (10 g/L of peptone140, 5 g/L of yeast extract, 10 g/L of NaCl pH 7.0) containing 50 μg/ml of kanamycin and 20 μM acetosyringone for 16 h at 28 °C/250 rpm. Bacterial cultures were suspended to a final O.D. of 0.5 per culture using MMA infiltration medium (20 g/L of sucrose, 5 g/L of Murashige and Skoog basal salt mixture, 1.95 g/L of 2-(*N*-morpholino)ethanesulfonic acid pH5.6) containing 200 μM acetosyringone. The *Agrobacterium* suspension was infiltrated into the youngest fully expanded leaves of 5–6 weeks old *Nicotiana benthamiana* plants at the abaxial side, which were then maintained in a controlled greenhouse compartment for 5–6 days (UNIFARM, Wageningen, Netherlands) prior to harvest.

### 2.2 Purification of BmVAL-1

BmVAL-1 was purified from the leaf extracellular space (apoplast) as described previously (Wilbers et al., 2017). Briefly, the infiltrated leaves were submerged in ice-cold extraction buffer (20 mM sodium phosphate buffer pH 6, 100 mM NaCl and 0.1% (v/v) Tween-20). After vacuum infiltration of the submerged leaves, apoplast fluid was retrieved by centrifugation (10 min at 2000*g*) and clarified by centrifugation (5 min at 16,000 *g*). BmVAL-1 was purified from the apoplast fluid using HS POROS 50 strong cation exchange (CEX) resin (Applied Biosystems, USA). Prior to purification, the apoplast fluid was passed over a G25 sephadex column with CEX binding buffer (20 mM sodium phosphate buffer pH 6, 100 mM NaCl). BmVAL-1 bound to CEX resin was eluted with 20 mM Tris-HCl buffer pH 9.0 containing 2 M NaCl. The purification was performed on a ÄKTA Prime Chromatography System (GE Healthcare, USA) using a constant flow rate of 10 mL/min for binding and washing and 2 mL/min for elution. Eluted BmVAL-1 was dialyzed overnight into PBS. Recombinant BmVAL-1 was separated under reduced conditions by SDS-PAGE on a 12% Bis-Tris gel (Invitrogen, USA) and subsequently stained with Coomassie brilliant blue staining.

### 2.3 Analysis of N-glycan composition

For N-glycan analysis, 1–2 μg of purified BmVAL-1 was reduced and denatured for 10 min at 95 °C in PBS containing 1.3% (w/v) SDS and 0.1% (v/v) β-mercaptoethanol. SDS was neutralized by adding 2% (v/v) NP-40 prior to overnight digestion at 37 °C with trypsin (Sigma-Aldrich, USA), immobilized to N-hydroxysuccinimide-activated sepharose (GE Healthcare). Trypsin beads were removed from the digestion mix by centrifugation and the pH of the mix was adjusted to 5 using 1 M sodium acetate. PNGase A (0.5 mU; Roche, Switzerland) was used to release N-glycans from BmVAL-1 while incubating overnight at 37 °C. The incubation mixture was applied to C18 Bakerbond™ SPE cartridges (JT Baker, USA) and the N-glycans were extracted from the flow-through on Extract Clean™ Carbo SPE columns. Eluted N-glycans were labeled with anthranilic acid (Sigma-Aldrich) and desalted by hydrophilic interaction chromatography on Biogel P10 (BioRad, USA). Samples in 75% acetonitrile were mixed with 1 μl of matrix solution (20 mg/ml 2,5-dihydroxybenzoic acid in 50% (v/v) acetonitrile, 0.1% (v/v) trifluoroacetic acid) and were dried under a stream of warm air. Matrix-assisted laser desorption/ionization (MALDI) time-of-flight mass spectra (MS) were obtained using an Ultraflex II mass spectrometer (Bruker Daltonics, USA).

### 2.4 Crystallization and structure determination

BmVAL-1 (10 mg/ml) in PBS was screened for crystallization conditions with commercial screens from Qiagen (Germany) and Microlytics (USA). Similar to human and parasite CAP proteins, BmVAL-1 crystals grew in high concentrations of polyethylene glycol. The best diffracting crystals were optimized by vapor diffusion in sitting drops by mixing 2.5 μL of protein solution with 1.5 μL of the precipitant (25% (w/v) PEG 4000, 0.2 M lithium sulfate, 0.1 M sodium acetate, and 0.1 M HEPES pH 7.5) at 298 K.

A single crystal was flash-cooled directly in a stream of N_2_ gas at 113 K to facilitate collecting diffraction data at the Baylor College of Medicine, USA, core facility using a Rigaku HTC detector. The X-ray source was a Rigaku FR-E+ SuperBright microfocus rotating anode generator with VariMax HF optics. Data was collected at a crystal-to-detector distance of 115 mm, with exposure times of 60 s for 0.5° oscillations, using the Crystal Clear (d∗trek) package (Pflugrath, 1999) and processed using MosFLM (Leslie, 2006). The molecular replacement phases were calculated with PHASER (McCoy et al., 2007) using Na-ASP-2 stripped of water and its carboxyl terminus extension as the search model. The resulting structural model was improved through automatic model building with ARP/wARP (Morris et al., 2003, Morris et al., 2004) followed by iterative manual model building cycles using the program Coot (Emsley et al., 2010), followed by structure refinement with both REFMAC5 (Murshudov et al., 2011) within the CCP4 package (Winn et al., 2011) and Phenix (Terwilliger et al., 2008, Adams et al., 2010). Ribbon diagram and model figures were generated using PyMOL. Data collection and structure refinement details are reported in Table 1. The atomic coordinates and structure factors for BmVAL-1 have been deposited in the protein data bank under accession number 6ANY. CAVER 3.0 analysis was performed within PyMOL (www.pymol.org) using default settings and centering in different positions in proximity to known cavities (Petrek et al., 2006, Chovancova et al., 2012, Kozlikova et al., 2014, Pavelka et al., 2016)

**Table 1:** Data collection, and refinement statistics for *Brugia malayi* venom allergen-like 1 protein (BmVAL-1).

Data collection	BmVAL-1
Wavelength (nm)	0.15418
Resolution range (Å)	44.87–2.25 (2.33–2.25)
Space group	*P* 4_3_ 2_1_ 2
Unit cell	*a* = 85.79 Å, *b* = 85.79 Å, *c* = 66.67 Å *α* = *β* = *γ* = 90^o^
Total reflections	22,409 (1735)
Unique reflections	11,799 (997)
Multiplicity	1.9 (1.7)
Completeness (%)	95.81 (83.07)
Mean I/sigma (I)	10.20 (3.50)
Wilson B-factor	20.07
*R-merge*	0.03196 (0.1561)
*R-meas*	0.0452 (0.2207)
*R-pim*	0.03196 (0.1561)
CC1/2	0.998 (0.927)
CC^*^	0.999 (0.981)
Reflections used in refinement	11,789 (996)
Reflections used for R-free	976 (72)
R-work	0.1823 (0.2052)
R-free	0.2134 (0.2755)
CC (work)	0.945 (0.853)
CC (free)	0.917 (0.740)
Number of non-hydrogen atoms	1882
Macromolecules	1630
Ligands	47
Solvent	205
Protein residues	206
RMSD bond lengths (Å)	0.008
RMSD angles (^o^)	0.93
Ramachandran favored (%)	99
Ramachandran allowed (%)	1.5
Ramachandran outliers (%)	0
Rotamer outliers (%)	0
Clashscore	6.01
Average B-factor	22.15
Macromolecules	20.86
Ligands	39.48
Solvent	28.45

Statistics for the highest resolution shell are shown in parentheses.

RMSD, root-mean-square deviation; CC, correlation coefficient.

### 2.5 In vivo sterol export from mutant yeast cells

Acetylation and export of sterols into the culture supernatant was examined as described (Tiwari et al., 2007). Heme (*hem1Δ*) -deficient yeast cells were cultivated in presence of Cholesterol/Tween 80 containing media and labeled with 0.025 µCi/ml [^14^C] cholesterol (American Radiolabeled Chemicals Inc, St. Louis, MO, USA). Cells were harvested by centrifugation, washed twice with synthetic complete (SC) media, diluted to an O.D._600_ of 1 into fresh media containing non-radiolabeled cholesterol and grown overnight. Cells were centrifuged and lipids were extracted from the cell pellet and the culture supernatant using chloroform/methanol (v/v 1:1). Samples were dried and separated by thin-layer chromatography (TLC) using silica gel 60 plates (Merck, Darmstadt, Germany) using the solvent system, petroleum ether/diethyl ether /acetic acid (70:30:2; per vol.). Radiolabeled lipids on the TLC were visualized and quantified by phosphorimaging.

### 2.6 In vitro lipid binding

Lipid binding was assessed in vitro using a radioligand-binding assay as described previously (Choudhary and Schneiter, 2012, Im et al., 2005). To measure sterol binding, 100 pmol of purified protein in binding buffer (20 mM Tris, pH 7.5, 30 mM NaCl, 0.05% Triton X-100) were incubated with 0–500 pmol of [^3^H]-cholesterol (American Radiolabeled Chemicals Inc., St Louis, Missouri, USA) for 1 h at 30 °C. The protein was then separated from the unbound ligand by adsorption to Q-sepharose beads (GE healthcare, USA), beads were washed, and the radioligand was quantified by scintillation counting. The effect of divalent cations on cholesterol binding was measured by performing the in vitro binding reaction in the presence of different concentrations of EDTA and magnesium chloride. At least two independent experiments were performed under each experimental condition and data is reported as the mean ± S.D. Calculation of the *K_d_* value and curve fitting were performed using the statistical software Prism, (GraphPad, La Jolla, CA, USA).

Similarly, to measure palmitate binding, purified proteins (100 pmol) in binding buffer (20 mM Tris pH 7.5, 30 mM NaCl, 0.05% Triton X-100) were incubated with [^3^H]-palmitic acid (0–400 pmol) for 1 h at 30 °C. The protein was then separated from the unbound ligand by adsorption to Q-sepharose beads (GE Healthcare); beads were washed with washing buffer (20 mM Tris pH 7.5), proteins were eluted (20 mM Tris pH 7.5, 1 M NaCl), and the radioligand was quantified by scintillation counting. To determine non-specific binding, the binding reaction was performed without the addition of protein into the binding assay.

## 3 Results

### 3.1 Production and structure of BmVAL-1

**Fig. 1:**
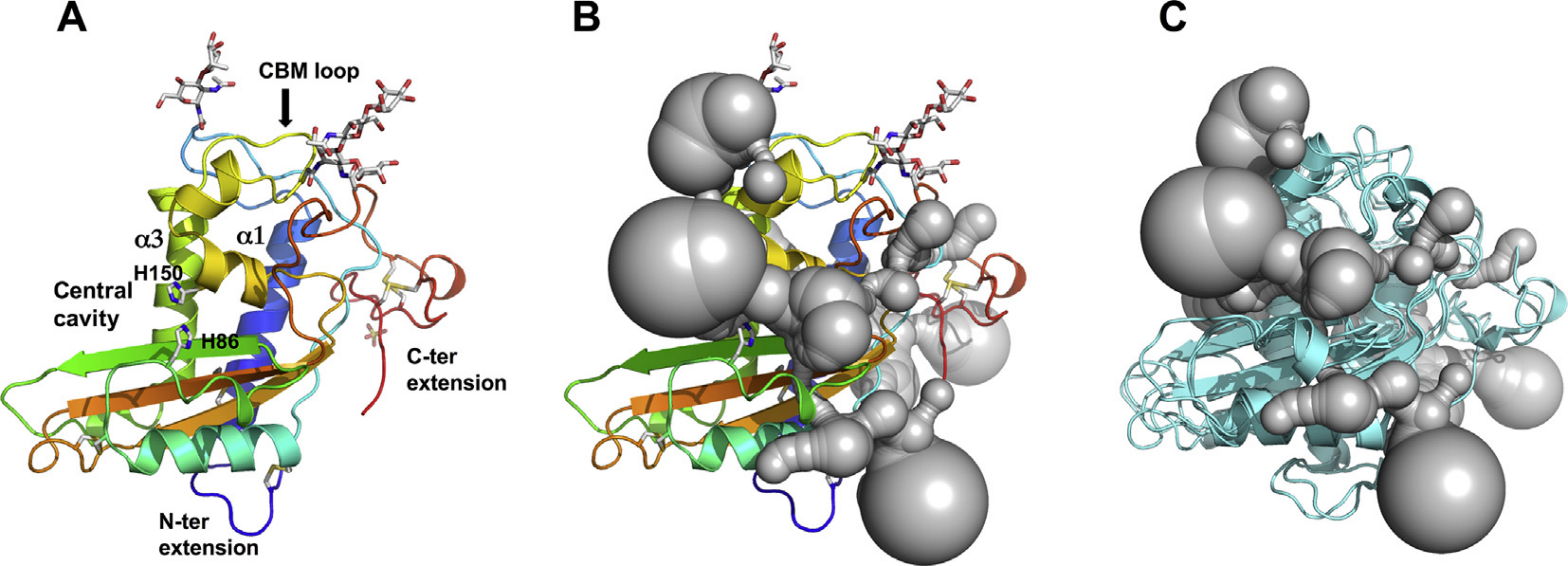
Structure of *Brugia malayi* venom allergen-like protein 1 (BmVAL-1). (A) Cartoon of a monomer of BmVAL-1 in rainbow colours from N-ter (blue) to C-ter (red). The two longest helices, α 1 and α3, that form the palmitate cavity and the caveolin binding motif loop (CBM) are indicated, while glycans are shown as sticks. Also shown are the histidines that coordinate divalent cations in the central cavity. (B) Channels generated with CAVER 3.0 (in gray) link all major cavities (shown as gray bubbles) on a monomer of BmVAL-1. (C) Superposition of *Necator americanus Ancylostoma* secreted protein-2 (Na-ASP-2), and pathogen-related yeast protein 1 (Pry1) with BmVAL-1 (shown as aquamarine cartoons) and the channels and cavities generated from BmVAL-1 with CAVER 3.0. B and C are shown in same view as A.

**Supplementary Fig. 1:**
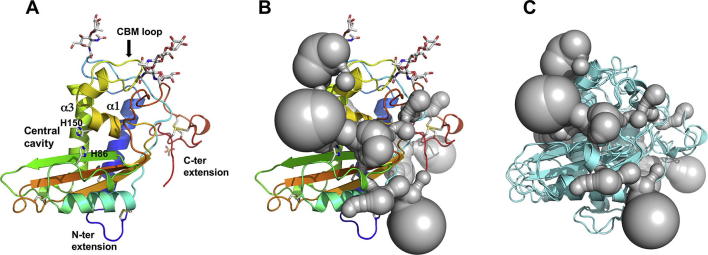


Recombinant BmVAL-1 was produced as a glycosylated protein using a plant expression system. A typical yield of 0.5–1.0 mg of pure recombinant protein was obtained per plant (3–4 g of leaf). Recombinant BmVAL-1 was shown to be ∼95% pure as assessed by Coomassie stained SDS-PAGE gel (Supplementary Fig. S1A). The two predicted N-linked glycosylation sites of BmVAL-1 (N52 and N138) are glycosylated and the branches have highly ordered *2Fo-Fc* electron density contoured at 1.5 sigma. The glycosylation in the electron density maps is consistent with the result from matrix-assisted laser desorption/ionization time-of-flight mass spectrometry (MALDI-TOF MS) analysis of released N-glycans. MALDI-TOF MS analysis shows that all N-glycan on BmVAL-1 have typical plant beta(1,2)-xylose and core alpha(1,3)-fucose residues. The majority of the N-glycans are paucimannosidic N-glycans (Supplementary Fig. S1B). Some N-glycans with one terminal GlcNAc residue (MGnXF^3^ or GnMXF^3^) were also detected. The glycosylation site at N138 is within the caveolin-binding motif (CBM) loop that is required for sterol export (Fig. 1A).

Secreted BmVAL-1 has 206 amino acid residues, two of which are vector-derived residues at the C-terminus. All 206 amino acid residues have ordered main and side chain electron density maps, of which 36 (17.5%) fold as beta strands, 59 (28.6%) fold as alpha helices, 11 (5.3%) fold as 3–10 helices, while 100 (48.5%) form loops. The overall topology of BmVAL-1 is an alpha-beta-alpha sandwich, in which a mixed strand beta sheet is sandwiched between two helical/loop regions. There are five disulfide bridges in the BmVAL-1 structure (19–69, 82–170, 164–180, 200–211 and 206–218). The last two disulfides stabilize a unique carboxyl terminus extension that is made mostly of loops. BmVAL-1 also has a loop on the amino terminus that forms a disulfide bridge with alpha helix-2. A single sulfate ion from the crystallization solution is present in the structure in proximity to the carboxyl terminus. The overall structure of BmVAL-1 is a prototypical CRISP-type SCP/TAPS protein that has two histidine residues which coordinate divalent cations located in the large central cavity (Fig. 1A).

The central cavity of BmVAL-1 has a volume of 2567 Å^3^ which is larger than other single CAP-domain SCP/TAPS protein structures including Na-ASP-2 (1824 Å^3^), Pry1^CAP^ (1591 Å^3^), GLIPR-1 (2000 Å^3^), GAPR-1 (1303 Å^3^), ves v5 (2048 Å^3^) and tablysin-15 (2263 Å^3^) as assessed by PDBsum (Henriksen et al., 2001, Serrano et al., 2004, Asojo et al., 2005, Asojo et al., 2011, Ma et al., 2011, de Beer et al., 2014, Darwiche et al., 2016, Laskowski et al., 2018). CAVER 3.0 analyses reveal that the central cavity extends around the monomer from the carboxyl terminus and is connected to the palmitate-binding cavity, CBM and carboxyl terminus loop with channels (Fig. 1B). These channels are large enough to allow small molecules such as water, ions and small ligands to pass between cavities, but too small to fit molecules the size of palmitate or cholesterol. We believe this is the first report of the connection of these cavities to form transport channels within a single CAP domain, however, channels had been previously reported in the Pry1^CAP^ dimer (Darwiche et al., 2016) and the two-CAP domain Na-ASP-1 (Asojo, 2011). The CBM and central cavities are separate and distinct cavities and the channels allow divalent cations bound in the central cavity to access the CBM. Superposing the structures of CRISP-type SCP/TAPS that have confirmed in vitro sterol binding reveals that similar channels can be formed in these proteins (Fig. 1C).

### 3.2 BmVAL-1 binds and exports cholesterol

**Fig. 2:**
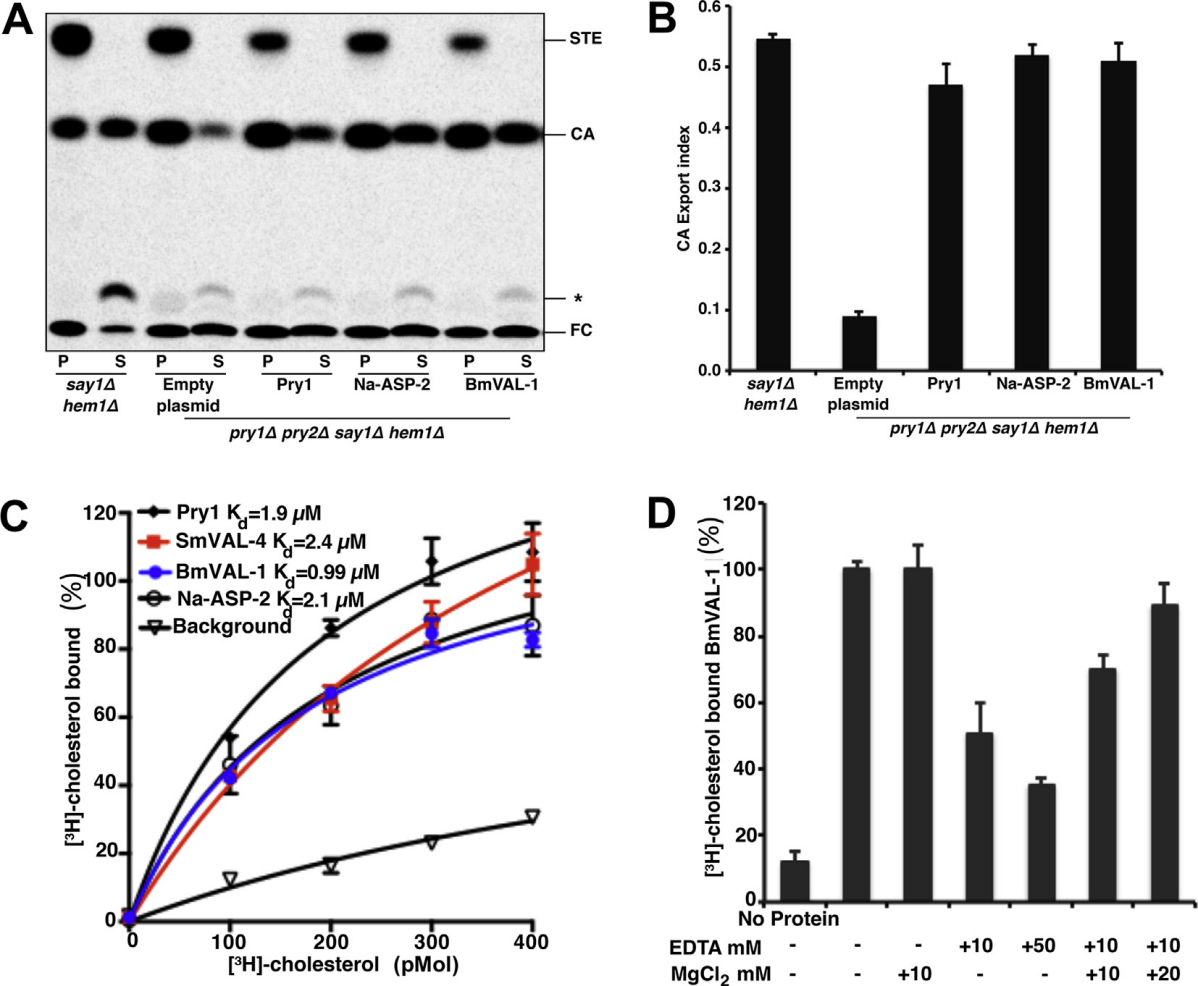
In vivo and in vitro sterol binding by *Brugia malayi* venom allergen-like protein 1 (BmVAL-1). (A) Expression of BmVAL-1, *Necator americanus Ancylostoma* secreted protein-2 (Na-ASP-2) and pathogen-related yeast protein 1 (Pry1) complement the sterol export defect of yeast cells lacking their endogenous Pry1,2. Heme-deficient cells of the indicated genotype containing either an empty plasmid or a plasmid with BmVAL-1, Na-ASP-2 or Pry1 were radiolabeled with [^14^C]cholesterol overnight, washed and diluted in fresh media to allow for export of cholesterol and cholesteryl acetate. Lipids were extracted from the cell pellet (P) and the culture supernatant (S), and separated by thin layer chromatography. The positions of free cholesterol (FC), cholesteryl acetate (CA) and steryl esters (STE) are indicated. The star marks the position of an unidentified cholesterol derivative. (B) Quantification of the export of cholesteryl acetate. The export index indicates the relative percentages of cholesteryl acetate that are exported by the cells (ratio between the extracellular cholesteryl acetate and the sum of intra- and extra-cellular cholesteryl acetate). Data represent mean ± S.D. of two independent experiments. (C) In vitro sterol binding by BmVAL-1 is comparable with Pry1, *Schistosoma mansoni* venom allergen-like protein 4 (SmVAL-4) and Na-ASP-2. (D) Addition of MgCl_2_ rescues the loss in sterol transport caused by the addition of EDTA.

Since BmVAL-1 has a cholesterol-binding CBM cavity, its ability to bind sterol in vitro and export sterol in vivo were assessed. BmVAL-1 is functional for cholesteryl acetate export in vivo and a plasmid encoding BmVAL-1 was able to restore the cholesteryl acetate export defect of mutant yeast cells that lacked endogenous Pry1 and Pry2 (Fig. 2A). BmVAL-1 has a comparable sterol export index to Pry1 (Fig. 2B). Addition of an increasing amount of [^3^H]-cholesterol resulted in a concentration-dependent and saturable binding of cholesterol to recombinant BmVAL-1. BmVAL-1 displayed saturation binding kinetics with an apparent *K_d_* of 0.99 µM, which is comparable with cholesterol binding by Pry1, Na-ASP-2, and *Schistosoma mansoni* venom allergen-like protein 4 (SmVAL-4), which have *K_d_* of 1.9 µM, 2.1 µM and 2.4 µM, respectively (Fig. 2C). Since BmVAL-1 is a CRISP type SCP/TAPS, having two histidines (H86 and H150) that are capable of coordinating divalent cations, the effect of divalent cations on sterol binding was determined. As observed for Pry1, EDTA inhibits cholesterol binding by BmVAL-1, and adding magnesium ions restores sterol binding, indicating that magnesium is important for sterol binding by BmVAL-1 (Fig. 2D).

### 3.3 Lipid binding by BmVAL-1

**Fig. 3:**
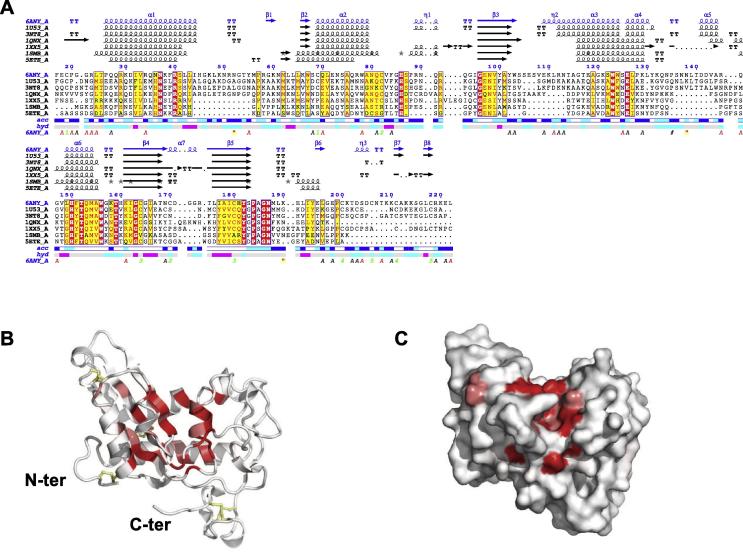
Structural alignment of *Brugia malayi* venom allergen-like protein 1 (BmVAL-1) with selected SCP/TAPS (Sperm-coating protein/Tpx/antigen 5/pathogenesis related-1/Sc7) proteins. (A) ENDscript (Gouet et al., 2003, Robert and Gouet, 2014) alignment identifies conserved residues in CRISP-type SCP/TAPS proteins. Amino acid numbering corresponds to full-length BmVAL-1. Also shown are vector-derived C-ter amino acid residues. Identical and conserved residues are highlighted in red and yellow, respectively. The different secondary structure elements shown are alpha helices (α), 3_10_-helices (η), beta strands (β), and beta turns (TT). The representative structural models with their respective protein data bank codes are Na-ASP-2 (1U53); Na-ASP-1 (3NT8); GAPR-1 (1SMB) (van Galen et al., 2012); a major allergen from Vespula vulgaris venom, Ves v 5, (1QNX), (Henriksen et al., 2001); the snake venom protein natrin, 1XX5, (Wang et al., 2006); and Pry1 CAP domain 5ETE (Darwiche et al., 2016). Solvent accessibility (acc) and hydropathy scales per residue (hyd) are also indicated. (B) Ribbon and (C) surface plots of BmVAL-1 reveal that identical residues cluster mostly around the central cavity. Identical residues are shown in red while conserved are shown in pink, and disulfide bridges are shown in yellow.

**Fig. 4:**
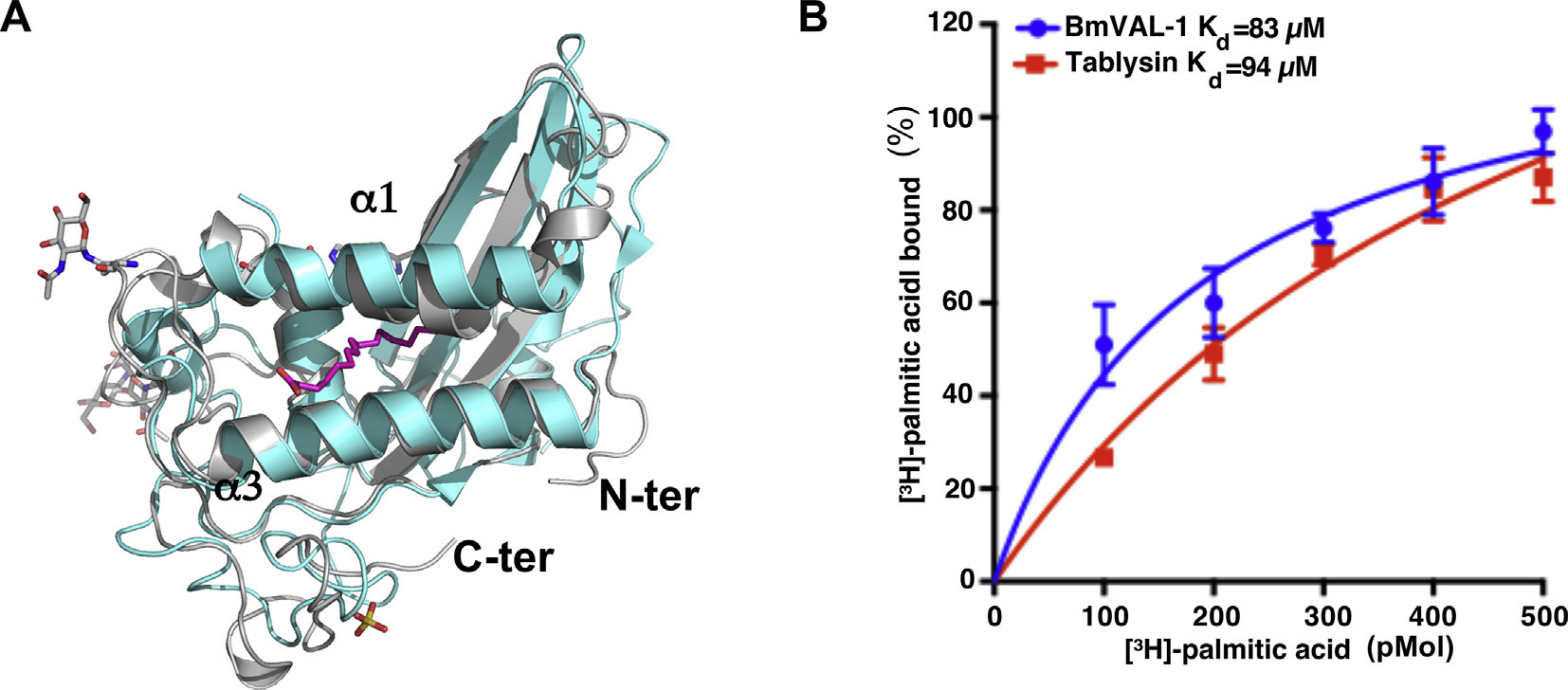
Palmitate binding by *Brugia malayi* venom allergen-like protein 1 (BmVAL-1). (A) Alignment of the palmitate binding cavities of BmVAL-1 (gray) and tablysin-15 (aquamarine); palmitate is shown in magenta. (B) In vitro palmitate binding activity of BmVAL-1 is comparable with tablysin-15.

The ability to bind palmitate is based upon the presence of the large cavity between two helices, as observed in tablysin-15, that was also shown to bind leukotriene (Xu et al., 2012). These central long alpha helices are present in SCP/TAPS proteins including BmVAL-1 (Fig. 3, Fig. 4A). As previously indicated in other CAP proteins, the amino acid residues in the palmitate-binding cavity are poorly conserved, however there is sufficient space between the equivalent helices to facilitate binding palmitate or similar lipids. The binding was confirmed using the in vitro palmitate-binding assay and the *K_d_* of BmVAL-1 (83 µM) is comparable with tablysin-15 (*K_d_* of 94 µM), which is a known palmitate binder (Fig. 4B). These analyses reveal that BmVAL-1 is structurally able to bind palmitate, as was observed for tablysin-15.

## 4 Discussion

BmVAL-1 is the first structure of a SCP/TAPS protein with a glycosylated CBM loop that exports sterols, which indicates that glycosylation of the CBM does not inhibit sterol binding. The most similar structure to BmVAL-1 was identified with PDBeFold (http://www.ebi.ac.uk/msd-srv/ssm/) as the molecular replacement search model Na-ASP-2 which only shares 35% sequence identity. Similar to Na-ASP-2, BmVAL-1 has an overall topology of a three-layered alpha-beta-alpha sandwich flanked by an amino terminal loop and a cysteine rich carboxyl terminal loop (Fig. 3, Fig. 4). However the loops in BmVAL-1 are oriented differently than in other SCP/TAP structures and the helices and strands are of different lengths than previously reported. The two disulfide bridges that stabilize the carboxyl terminal extension are located in similar positions as in other nematode SCP/TAPS structures despite conformational differences in these extensions (Asojo et al., 2005, Asojo, 2011, Borloo et al., 2013, Mason et al., 2014). A similar carboxyl terminal extension was shown serving as a linker domain in snake venom CRISPs (Asojo et al., 2005). The possible roles of these carboxyl terminal extensions are unknown, however this extension is not necessary for sterol binding or transport, as proteins lacking this extension (Pry1CAP, SmVAL-4) are capable of in vivo and in vitro sterol binding.

BmVAL-1 has a much larger central cavity than previously observed in other SCP/TAPS proteins and for the first time the interconnectivity of the central cavity to other cavities via channels was observed in a monomer. The observation that similar channels may connect the different binding sites and the central cavity explains the ability of seemingly independent cavities to affect each other, specifically that blocking divalent ions in the central cavity affects sterol binding in the distinct CBM cavity. The channels are quite small and while they may allow passage of ions, water, and possibly small ligands within the monomer, the channels are not large enough for cholesterol or palmitate.

**Supplementary Fig. 2:**
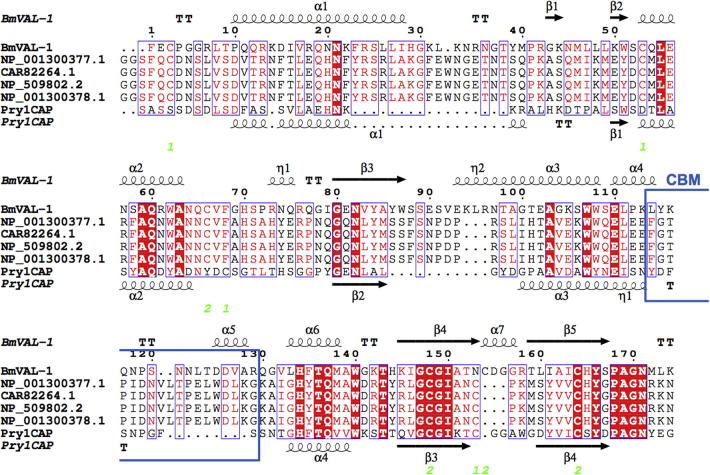


While our current work offers insights into cholesterol and palmitate binding by BmVAL-1, questions remain about how this affects the roles of the protein in the parasite as well as its use as a potential vaccine. It is known that SCP/TAPS proteins are predominant proteins secreted during the first molt of both infective and free-living nematodes. Furthermore, cholesterol and its derivatives have been shown to be important in molting in *Caenorhabditis elegans* (Entchev and Kurzchalia, 2005). The CAP domain, and more specifically the sterol-binding CBM loops, of *C. elegans* VALs are similar to BmVAL-1, suggesting similar binding properties to BmVAL-1 (Supplementary Fig. S2). More studies are required to determine if sterol binding by SCP/TAPS proteins affects the transition to parasitism in parasitic nematodes and to identify possible roles of VALs in nematode molting. The binding and sequestration of small molecules such as cholesterol and fatty acids may affect the vaccine efficacy of BmVAL-1, and it remains to be determined whether BmVAL-1 has the ability to bind commonly used adjuvants such as squalene.

With the crystal structure of BmVAL-1 now revealed, a more detailed antigenic analysis of this vaccine candidate will be possible. In particular, the structure defines exposed regions which may be epitopes preferentially recognized by antibodies in infected subjects and/or vaccinated animals, and can serve as a starting point to define features common to VAL antigens from different helminth species, as well as those unique to BmVAL-1 which may confer upon it protective potential against filarial infection.

The structure of the first filarial nematode SCP/TAPS protein BmVAL-1 reveals structural similarity to hookworm Na-ASP-2. Uniquely, BmVAL-1 has a larger central cavity and a glycosylated CBM with uncompromised sterol-binding ability. While sterol is required for molting, the roles of SCP/TAPS proteins in molting require further investigation. The structure of BmVAL-1 reveals for the first known time that the central, sterol-binding CBM, and palmitate-binding cavities are connected within a monomer by tunnels that are large enough for the passage of ions and water molecules, which may explain how sterol binding is affected by divalent cations bound in distinct cavities. Future studies will clarify the roles of the carboxyl terminal extension as well as identify lipids that specifically bind to the palmitate-binding cavity. There is also a need to determine whether the ability to bind so many small molecules will affect the possible application of CAP proteins as vaccines.

## References

[b0005] Adams P.D., Afonine P.V., Bunkoczi G., Chen V.B., Davis I.W., Echols N., Headd J.J., Hung L.W., Kapral G.J., Grosse-Kunstleve R.W., McCoy A.J., Moriarty N.W., Oeffner R., Read R.J., Richardson D.C., Richardson J.S., Terwilliger T.C., Zwart P.H. (2010). PHENIX: a comprehensive Python-based system for macromolecular structure solution. Acta Crystallogr. D.

[b0010] Anand S.B., Gnanasekar M., Thangadurai M., Prabhu P.R., Kaliraj P., Ramaswamy K. (2007). Immune response studies with *Wuchereria bancrofti* vespid allergen homologue (WbVAH) in human lymphatic filariasis. Parasitol. Res..

[b0015] Asojo O.A. (2011). Crystal Structure of a two-CAP domain protein from the human hookworm parasite *Necator americanus*. Acta Crystallogr. D.

[b0020] Asojo O.A., Goud G., Dhar K., Loukas A., Zhan B., Deumic V., Liu S., Borgstahl G.E., Hotez P.J. (2005). X-ray structure of Na-ASP-2, a pathogenesis-related-1 protein from the nematode parasite, *Necator americanus*, and a vaccine antigen for human hookworm infection. J. Mol. Biol..

[b0025] Asojo O.A., Koski R.A., Bonafe N. (2011). Structural studies of human glioma pathogenesis-related protein 1. Acta Crystallogr. D.

[b0030] Borloo J., Geldhof P., Peelaers I., Van Meulder F., Ameloot P., Callewaert N., Vercruysse J., Claerebout E., Strelkov S.V., Weeks S.D. (2013). Structure of *Ostertagia ostertagi* ASP-1: insights into disulfide-mediated cyclization and dimerization. Acta Crystallogr. D.

[b0035] Choudhary V., Schneiter R. (2012). Pathogen-Related Yeast (PRY) proteins and members of the CAP superfamily are secreted sterol-binding proteins. Proc. Natl. Acad. Sci. U.S.A..

[b0040] Chovancova E., Pavelka A., Benes P., Strnad O., Brezovsky J., Kozlikova B., Gora A., Sustr V., Klvana M., Medek P., Biedermannova L., Sochor J., Damborsky J. (2012). CAVER 3.0: a tool for the analysis of transport pathways in dynamic protein structures. PLoS Comput. Biol..

[b0045] Darwiche R., El Atab O., Cottier S., Schneiter R. (2017). The function of yeast CAP family proteins in lipid export, mating, and pathogen defense. FEBS Lett..

[b0050] Darwiche R., Kelleher A., Hudspeth E.M., Schneiter R., Asojo O.A. (2016). Structural and functional characterization of the CAP domain of pathogen-related yeast 1 (Pry1) protein. Sci. Rep..

[b0055] de Beer T.A., Berka K., Thornton J.M., Laskowski R.A. (2014). PDBsum additions. Nucleic Acids Res..

[b0060] de Castro E., Sigrist C.J., Gattiker A., Bulliard V., Langendijk-Genevaux P.S., Gasteiger E., Bairoch A., Hulo N. (2006). ScanProsite: detection of PROSITE signature matches and ProRule-associated functional and structural residues in proteins. Nucleic Acids Res..

[b0065] Ding X., Shields J., Allen R., Hussey R.S. (2000). Molecular cloning and characterisation of a venom allergen AG5-like cDNA from *Meloidogyne incognita*. Int. J. Parasitol..

[b0070] Emsley P., Lohkamp B., Scott W.G., Cowtan K. (2010). Features and development of Coot. Acta Crystallogr. D.

[b0075] Entchev E.V., Kurzchalia T.V. (2005). Requirement of sterols in the life cycle of the nematode *Caenorhabditis elegans*. Semin. Cell Dev. Biol..

[b0080] Gamir J., Darwiche R., Van't Hof P., Choudhary V., Stumpe M., Schneiter R., Mauch F. (2017). The sterol-binding activity of Pathogenesis-Related protein 1 reveals the mode of action of an antimicrobial protein. Plant J..

[b0085] Gao B., Allen R., Maier T., Davis E.L., Baum T.J., Hussey R.S. (2001). Molecular characterisation and expression of two venom allergen-like protein genes in *Heterodera glycines*. Int. J. Parasitol..

[b0090] Gibbs G.M., Roelants K., O'Bryan M.K. (2008). The CAP superfamily: cysteine-rich secretory proteins, antigen 5, and pathogenesis-related 1 proteins roles in reproduction, cancer, and immune defense. Endocr. Rev..

[b0095] Gouet P., Robert X., Courcelle E. (2003). ESPript/ENDscript: Extracting and rendering sequence and 3D information from atomic structures of proteins. Nucleic Acids Res..

[b0100] Hawdon J.M., Narasimhan S., Hotez P.J. (1999). *Ancylostoma* secreted protein 2: cloning and characterization of a second member of a family of nematode secreted proteins from *Ancylostoma caninum*. Mol. Biochem. Parasitol..

[b0105] Henriksen A., King T.P., Mirza O., Monsalve R.I., Meno K., Ipsen H., Larsen J.N., Gajhede M., Spangfort M.D. (2001). Major venom allergen of yellow jackets, Ves v 5: structural characterization of a pathogenesis-related protein superfamily. Proteins.

[b0110] Im Y.J., Raychaudhuri S., Prinz W.A., Hurley J.H. (2005). Structural mechanism for sterol sensing and transport by OSBP-related proteins. Nature.

[b0115] Kalyanasundaram R., Balumuri P. (2011). Multivalent vaccine formulation with BmVAL-1 and BmALT-2 confer significant protection against challenge infections with *Brugia malayi* in mice and jirds. Res. Rep. Trop. Med..

[b0120] Kazan K., Gardiner D.M. (2017). Targeting pathogen sterols: Defence and counterdefence?. PLoS Pathog..

[b0125] Kozlikova B., Sebestova E., Sustr V., Brezovsky J., Strnad O., Daniel L., Bednar D., Pavelka A., Manak M., Bezdeka M., Benes P., Kotry M., Gora A., Damborsky J., Sochor J. (2014). CAVER Analyst 1.0: graphic tool for interactive visualization and analysis of tunnels and channels in protein structures. Bioinformatics.

[b0130] Laskowski R.A., Jabonska J., Pravda L., Varekova R.S., Thornton J.M. (2018). PDBsum: Structural summaries of PDB entries. Protein Sci..

[b0135] Leslie A.G. (2006). The integration of macromolecular diffraction data. Acta Crystallogr. D.

[b0140] Li B.W., Rush A.C., Mitreva M., Yin Y., Spiro D., Ghedin E., Weil G.J. (2009). Transcriptomes and pathways associated with infectivity, survival and immunogenicity in *Brugia malayi* L3. BMC Genomics.

[b0145] Ma D., Xu X., An S., Liu H., Yang X., Andersen J.F., Wang Y., Tokumasu F., Ribeiro J.M., Francischetti I.M., Lai R. (2011). A novel family of RGD-containing disintegrins (Tablysin-15) from the salivary gland of the horsefly *Tabanus yao* targets alphaIIbbeta3 or alphaVbeta3 and inhibits platelet aggregation and angiogenesis. Thromb. Haemost..

[b0150] Mason L., Tribolet L., Simon A., von Gnielinski N., Nienaber L., Taylor P., Willis C., Jones M.K., Sternberg P.W., Gasser R.B., Loukas A., Hofmann A. (2014). Probing the equatorial groove of the hookworm protein and vaccine candidate antigen, Na-ASP-2. Int. J. Biochem. Cell. Biol..

[b0155] McCoy A.J., Grosse-Kunstleve R.W., Adams P.D., Winn M.D., Storoni L.C., Read R.J. (2007). Phaser crystallographic software. J. Appl. Crystallogr..

[b0160] Morris R.J., Perrakis A., Lamzin V.S. (2003). ARP/wARP and automatic interpretation of protein electron density maps. Methods Enzymol..

[b0165] Morris R.J., Zwart P.H., Cohen S., Fernandez F.J., Kakaris M., Kirillova O., Vonrhein C., Perrakis A., Lamzin V.S. (2004). Breaking good resolutions with ARP/wARP. J. Synchrotron Radiat..

[b0170] Murray J., Gregory W.F., Gomez-Escobar N., Atmadja A.K., Maizels R.M. (2001). Expression and immune recognition of *Brugia malayi* VAL-1, a homologue of vespid venom allergens and *Ancylostoma* secreted proteins. Mol. Biochem. Parasitol..

[b0175] Murshudov G.N., Skubak P., Lebedev A.A., Pannu N.S., Steiner R.A., Nicholls R.A., Winn M.D., Long F., Vagin A.A. (2011). REFMAC5 for the refinement of macromolecular crystal structures. Acta Crystallogr. D.

[b0180] Pavelka A., Sebestova E., Kozlikova B., Brezovsky J., Sochor J., Damborsky J. (2016). CAVER: algorithms for analyzing dynamics of tunnels in macromolecules. IEEE/ACM Trans. Comput. Biol. Bioinform..

[b0185] Petrek M., Otyepka M., Banas P., Kosinova P., Koca J., Damborsky J. (2006). CAVER: a new tool to explore routes from protein clefts, pockets and cavities. BMC Bioinformatics.

[b0190] Pflugrath J.W. (1999). The finer things in X-ray diffraction data collection. Acta Crystallogr. D.

[b0195] Robert X., Gouet P. (2014). Deciphering key features in protein structures with the new ENDscript server. Nucleic Acids Res..

[b0200] Schneiter R., Di Pietro A. (2013). The CAP protein superfamily: function in sterol export and fungal virulence. Biomol. Concepts.

[b0205] Serrano R.L., Kuhn A., Hendricks A., Helms J.B., Sinning I., Groves M.R. (2004). Structural analysis of the human Golgi-associated plant pathogenesis related protein GAPR-1 implicates dimerization as a regulatory mechanism. J. Mol. Biol..

[b0210] Suzuki N., Yamazaki Y., Brown R.L., Fujimoto Z., Morita T., Mizuno H. (2008). Structures of pseudechetoxin and pseudecin, two snake-venom cysteine-rich secretory proteins that target cyclic nucleotide-gated ion channels: implications for movement of the C-terminal cysteine-rich domain. Acta Crystallogr. D.

[b0215] Terwilliger T.C., Grosse-Kunstleve R.W., Afonine P.V., Moriarty N.W., Zwart P.H., Hung L.W., Read R.J., Adams P.D. (2008). Iterative model building, structure refinement and density modification with the PHENIX AutoBuild wizard. Acta Crystallogr. D.

[b0220] Tiwari R., Koffel R., Schneiter R. (2007). An acetylation/deacetylation cycle controls the export of sterols and steroids from *S. cerevisiae*. EMBO J..

[b0225] van Galen J., Olrichs N.K., Schouten A., Serrano R.L., Nolte-'t Hoen E.N., Eerland R., Kaloyanova D., Gros P., Helms J.B. (2012). Interaction of GAPR-1 with lipid bilayers is regulated by alternative homodimerization. Biochim. Biophys. Acta.

[b0230] Wang F., Li H., Liu M.N., Song H., Han H.M., Wang Q.L., Yin C.C., Zhou Y.C., Qi Z., Shu Y.Y., Lin Z.J., Jiang T. (2006). Structural and functional analysis of natrin, a venom protein that targets various ion channels. Biochem. Biophys. Res. Commun..

[b0235] Wang Y.L., Kuo J.H., Lee S.C., Liu J.S., Hsieh Y.C., Shih Y.T., Chen C.J., Chiu J.J., Wu W.G. (2010). Cobra CRISP functions as an inflammatory modulator via a novel Zn^2+^- and heparan sulfate-dependent transcriptional regulation of endothelial cell adhesion molecules. J. Biol. Chem..

[b0240] Wilbers R.H., Westerhof L.B., van Noort K., Obieglo K., Driessen N.N., Everts B., Gringhuis S.I., Schramm G., Goverse A., Smant G., Bakker J., Smits H.H., Yazdanbakhsh M., Schots A., Hokke C.H. (2017). Production and glyco-engineering of immunomodulatory helminth glycoproteins in plants. Sci. Rep..

[b0245] Winn M.D., Ballard C.C., Cowtan K.D., Dodson E.J., Emsley P., Evans P.R., Keegan R.M., Krissinel E.B., Leslie A.G., McCoy A., McNicholas S.J., Murshudov G.N., Pannu N.S., Potterton E.A., Powell H.R., Read R.J., Vagin A., Wilson K.S. (2011). Overview of the CCP4 suite and current developments. Acta Crystallogr. D.

[b0250] Xu X., Francischetti I.M., Lai R., Ribeiro J.M., Andersen J.F. (2012). Structure of protein having inhibitory disintegrin and leukotriene scavenging functions contained in single domain. J. Biol. Chem..

[b0255] Yoon S., Ebert J.C., Chung E.Y., De Micheli G., Altman R.B. (2007). Clustering protein environments for function prediction: finding PROSITE motifs in 3D. BMC Bioinformatics.

[b0260] Zhan B., Liu Y., Badamchian M., Williamson A., Feng J., Loukas A., Hawdon J.M., Hotez P.J. (2003). Molecular characterisation of the *Ancylostoma*-secreted protein family from the adult stage of *Ancylostoma caninum*. Int. J. Parasitol..

